# Designing sgRNAs for CRISPR-BEST base editing applications with CRISPy-web 2.0

**DOI:** 10.1016/j.synbio.2020.05.005

**Published:** 2020-06-13

**Authors:** Kai Blin, Simon Shaw, Yaojun Tong, Tilmann Weber

**Affiliations:** The Novo Nordisk Foundation Center for Biosustainability, Technical University of Denmark, Kgs. Lyngby, Denmark

**Keywords:** CRISPR, Base editor, sgRNA, Webserver, CRISPR-BEST, Genome editing

## Abstract

CRISPR/Cas9 systems are an established tool in genome engineering. As double strand breaks caused by the standard Cas9-based knock-out techniques can be problematic in some organisms, new systems were developed that can efficiently create knock-outs without causing double strand breaks to elegantly sidestep these issues. The recently published CRISPR-BEST base editor system for actinobacteria is built around a C to T or A to G base exchange. These base editing systems however require additional constraints to be considered for designing the sgRNAs. Here, we present an updated version of the interactive CRISPy-web single guide RNA design tool https://crispy.secondarymetabolites.org/that was built to support “classical” CRISPR and now also CRISPR-BEST workflows.

## 1 Introduction

The RNA-guided endonucleases associated with “clusters of regularly interspaced palindromic repeats” (CRISPR) evolved as a bacterial adaptive immune system, protecting against unwanted foreign DNA, e.g. from bacteriophages [1]. During the last few years, these bacterial immune systems have been the basis for many successful CRISPR-based genome editing tools [2] that nowadays are widely used in molecular biology and synthetic biology from bacteria to human cells [[3], [4], [5]].

While many different CRISPR systems exist in nature [6], the type II CRISPR system CRISPR-Cas9 from *Streptococcus pyogenes* is the most widely used genome engineering tool. The endonuclease Cas9 introduces DNA double strand breaks (DSBs) at positions complementary to a 20-nt spacer containing CRISPR RNA (crRNA) that in turn complexes with a trans-activating crRNA (tracrRNA). In genome engineering applications, an artificially fused crRNA:tracrRNA complex is called “single guide RNA” (sgRNA) used [7] for ease of handling. A functional sgRNA is composed of a 20-nt spacer and the following sgRNA scaffold.

In order to use a CRISPR/Cas9 based system for gene edits, suitable spacer sequences need to be identified on the target gene (protospacer) that should be knocked out. The 20-bp spacer needs to be directly upstream of a protospacer-adjacent motif (PAM). In the case of *S. pyogenes* Cas9, the PAM sequence is “5′-NGG-3’”. To avoid unwanted effects in other parts of the genome, it is desirable to pick a spacer sequence that does not match in other locations of the genome, even considering imprecise matches with one to three base pairs differing. To assist researchers with picking good spacers for constructing good sgRNAs in their non-model microbial genomes, we published the CRISPy-web sgRNA design tool [8] in 2016.

In the last four years, it has become evident that the DSBs can have detrimental side-effects and lead to genome instability in some organisms, such as streptomycetes [9]. To avoid these complications, we recently extended our actinomycete CRISPR toolkit [10] with the single-nucleotide–resolution genome editing system CRISPR-BEST [11]. CRISPR-BEST, which is based on the CRISPR base editor technology [12,13], can utilise one of two different deaminase enzymes along with other DNA repair enzymes native to the host to perform either C to T (CRISPR-cBEST) or A to G (CRISPR-aBEST) conversions in a narrow edit window. This allows precise edits that e.g. can change a regular amino acid codon to a STOP codon to inactivate a specific gene or pathway without the cellular stress of a DSB.

In order to design sgRNAs for CRISPR-BEST/base editor applications additional parameters have to be considered: depending on the deaminase, only cytidines/adenines within a defined editing window of 13–20 bases upstream of the PAM are considered for CRISPR-cBEST/aBEST, respectively. Furthermore, for most applications only base changes that lead either to amino acid substitutions or translational STOP signals are of interest. While tools like BE-Designer [14] already support designing sgRNAs for base editing workflows, they are usually limited to a set of pre-calculated genomes. To easily identify base editing sgRNAs in non-model organisms, we have extended the existing CRISPy-web with CRISPR-BEST annotations. As the addition of the base edit window detection logic significantly increased the runtime of the predictions, we also took the opportunity to re-work how the results are generated in the back-end analysis service. Additionally, all of CRISPy-web was converted to Python 3.

Here we present the improved and extended version 2 of CRISPy-web (https://crispy.secondarymetabolites.org/).

## 2 Design and implementation

CRISPy-web is a multi-component web service connected by a message queue. The main components are the analysis service, a web application programming interface (API) service, and the web user interface (UI). Additionally, CRISPy-web functionality is available in a standalone script to integrate into other analysis pipelines.

Both the UI and API services were extended to support the input of CRISPR-BEST parameters for edit window size and edit window location relative to the PAM, but otherwise are largely unchanged from the previous version, with the bulk of the changes happening in the analysis service.

CRISPy-web allows the user to upload a bacterial or fungal genome of interest. While it is possible to use the software directly on FASTA sequence data, it is strongly advised to use annotated sequences uploaded in Genbank format. When such annotation is provided, CRISPy-web, for example, allows searching for gene names, locus tags or protein IDs or finding base editor targets, which require annotated gene coordinates. For private genomes, where such annotation cannot directly be obtained from NCBI Genbank/RefSeq or other databases, it can be generated with most commonly used genome annotation software. Popular choices in the bacterial world would be Prokka [15] or RAST [16], for fungi it would be using Augustus [17] and subsequently converting the GFF3 output into GenBank format. If users are interested in engineering secondary metabolite biosynthetic gene clusters, pre-analyzing the data with antiSMASH [18] is recommended.

The first step in sgRNA detection is finding the PAM associated with the Cas enzyme in use. Then, sequences 13 bp upstream of the PAM are analysed for their uniqueness with the number of off-target matches reported for exact matches and matches with up to three mismatches. To quickly undertake this search, we have developed a Python library for NEARby MISmatch Search (nearmiss, https://github.com/secondarymetabolites/nearmiss). Nearmiss uses efficiently constructed suffix arrays with induced sorting [19] and pointer arithmetic in a C module to find PAMs in O(|anchor|⋅log|sequence|) and off-target matches in $O(a\;\cdot \;{\left(sw\right)}^{d}\;\cdot \;log\;|\;sequence\;|)$, where a is the number of anchors found, s is the size of the alphabet (4 for DNA), w is the window size, and d is the maximum number of mismatches allowed in the window.

Once sgRNA candidates have been detected, their suitability for use in a CRISPR-BEST base editing system is analysed. To adjust for different edit systems, both the edit window size and the edit window distance from the PAM are parameterised. When the edit window is found to overlap with a gene, the base exchange is simulated to observe which codons change. Base replacements that only cause conservative mutations are discarded.

All sgRNAs in the region specified by the user are reported back to the user interface, which can filter for only sgRNAs that can be used for editing or even just for sgRNAs that can be used to introduce STOP codons.

## 3 Example usage: introducing a STOP mutation into the actinorhodin gene cluster of *Streptomyces coelicolor*

**Fig. 1:**
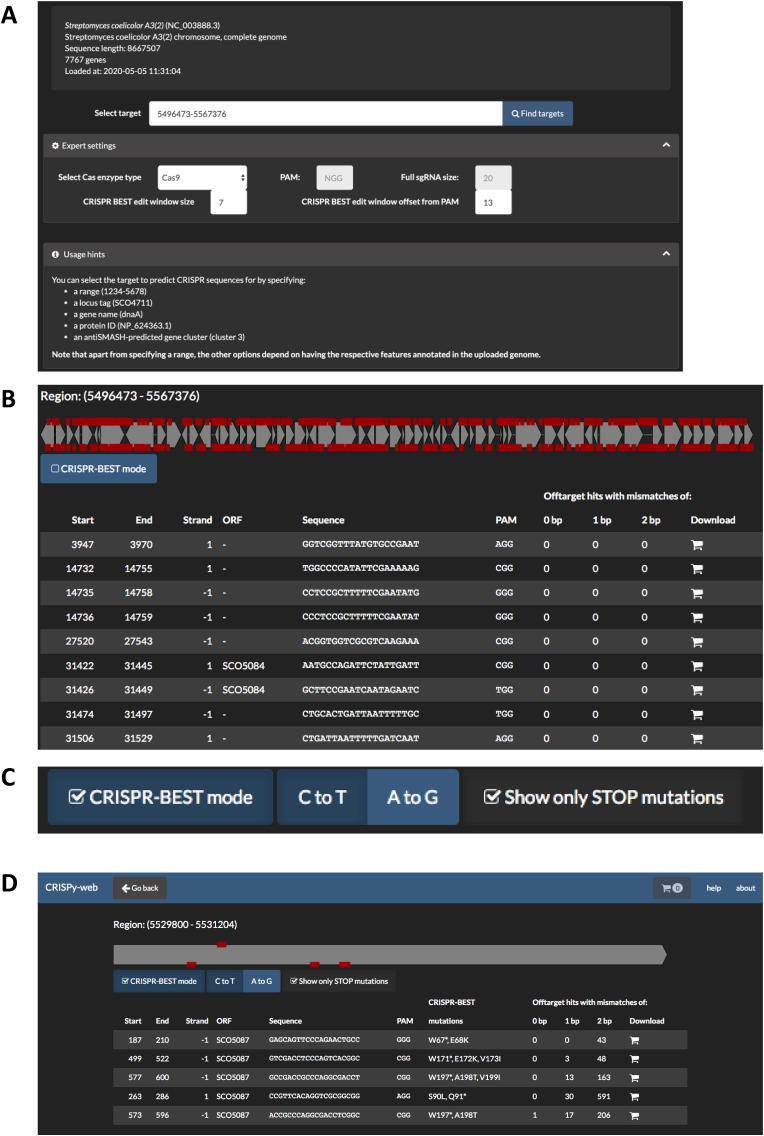
A: Target selection dialog with unfolded “Expert settings” to select the Cas enzyme (PAM and spacer size are filled out automatically) and base editor properties, and unfolded “Usage hints” describing the syntax to define the target sequence. B: Display of CRISPy-web sgRNA predictions for the actinorhodin-BGC of *S. coelicolor***.** C: Selection buttons to activate base editor/CRISPR-BEST mode. D: sgRNA predictions for CRISPR-cBEST base editing applications. Only sgRNAs are displayed that will lead to STOP mutations in the selected target gene (indicated by *); if there are further C bases within the editing window, all potential amino acid exchanges are displayed.

To be able to work on the actinorhodin cluster of *S. coelicolor*, the full genome GenBank file of *S. coelicolor* needs to be obtained from NCBI (RefSeq ID: NC_003888) and uploaded to CRISPy-web. After a quick scan of the uploaded genome, CRISPy-web displays the target selection screen. On the NC_003888 assembly, the actinorhodin cluster is located within a region spanning from 5,496,473–5,567,376 bp, so enter that base range on the target selection box (Fig. 1A). As the query sequence in the example contains full annotation from NCBI, it also would be possible to search for gene names, locus tag names or Protein IDs by entering these in the target selection box. Clicking “Find targets” starts the sgRNA search. Depending on the size of the genome and the size of the target region to scan for sgRNAs, the search can take several minutes.

Once the sgRNA search has completed, the target region will be displayed, with red boxes signifying potential sgRNAs (Fig. 1B). At the moment, these are all potential sgRNAs, regardless of their ability to cause STOP codons when used in the CRISPR-BEST system. Potential sgRNAs are sorted by the number of offtarget hits from that sgRNA in other areas of the genome, with 0, 1, or 2 mismatches in the 13 bp seed region right upstream of the PAM.

To narrow down the hits to only STOP codon introducing sgRNAs, select the “CRISPR-BEST mode” button. New buttons will appear to select “C to T” and “A to G” mode, with “C to T” mode being selected by default (Fig. 1C). This will reduce hits to sgRNAs that cause an amino acid change when used with CRISPR-cBEST. Clicking the “Show only STOP mutations” will further narrow down the number of sgRNA hits to only those that will cause STOP codons (Fig. 1D).

As there are still a lot of hits for the region, it is possible to zoom in on an individual gene you might want to knock out specifically by clicking on the respective gene arrow and selecting “Show results for this gene only”.

To download the sequences in a CSV file format, select the sgRNAs of interest by clicking on them, and then click on the shopping cart icon on the upper right. This takes you to the download overview page. Clicking the “Download CSV file” button initiates the download.

## CRediT authorship contribution statement

**Kai Blin:** Conceptualization, Methodology, Software, Writing - original draft, Writing - review & editing. **Simon Shaw:** Software. **Yaojun Tong:** Methodology, Validation. **Tilmann Weber:** Conceptualization, Supervision, Writing - review & editing.
